# Adherence to Usability and Accessibility Principles in Digital Health Applications for Patients With Diabetes: Systematic Review

**DOI:** 10.2196/71567

**Published:** 2025-09-26

**Authors:** Sarah Louise Watson, Hanan Khalid Mofty, Michael Donnelly, Tunde Peto, Ruth Esther Hogg

**Affiliations:** 1 Centre for Public Health School of Medicine, Dentistry and Biomedical Sciences Queen's University Belfast Belfast United Kingdom; 2 Department of Optometry and Vision Science College of Applied Medical Science King Saud University Riyadh Saudi Arabia

**Keywords:** accessibility, adherence, diabetes apps, digital health guidelines, reliability, usability

## Abstract

**Background:**

Health apps have the potential to enable people with diabetes to access care more easily, monitor their condition, and reduce the number of times they need to attend health care appointments. However, the development pipeline for apps may differ widely before the apps are released for use, due to limited funding, difficulty in obtaining iterative feedback from patients/users, and varying levels of developer expertise. In response to concerns about the quality and consistency of apps being released, two guidelines were created: the Digital Technology Assessment Criteria (DTAC) and the National Institute for Health and Care Excellence (NICE) Evidence Standards Framework. These two frameworks aim to standardize the development and evaluation of digital health technologies (DHTs). They outline core requirements, such as accessibility, clinical safety, data protection, interoperability, usability, and safeguarding, which help ensure that digital health apps are accessible, safe, effective, and suitable for real-world use.

**Objective:**

This systematic review evaluated the performance of diabetes digital health apps, as presented in published studies, in terms of adherence to DTAC 2021 and NICE 2022 guidelines during development.

**Methods:**

We systematically searched Embase and MEDLINE and identified 43 studies that met the inclusion criteria. Each study was assessed against 13 binary scoring criteria derived from the two frameworks.

**Results:**

Our findings highlighted that 93% (n=40) of the studies met fewer than 40% of the recommended criteria. Specifically, 88.4% (n=38) studies did not report accurate and reliable measurements, 86% (n=37) omitted app accuracy validation, and 83.7% (n=36) failed to address inequalities considerations. Only 3 (7%) studies achieved scores between 7 and 9 out of a possible 13, and none fully adhered to the guideline criteria.

**Conclusions:**

These results suggest a significant gap between digital health guidelines and real-world app development practices. We recommend the adoption of DTAC and NICE guidelines more widely and consistently during design and development. Additionally, we suggest that journals request that authors submit an adherence checklist alongside their manuscript to improve standardization and transparency across digital health publications.

**Trial Registration:**

PROSPERO CRD42022322040; https://www.crd.york.ac.uk/PROSPERO/view/CRD42022322040

## Introduction

Diabetes is an increasingly common health problem that affects 537 million adults worldwide [1]. Patients with this chronic condition are at increased risk of developing diabetic complications, such as diabetic nephropathy (DN), diabetic foot (DF), or diabetic retinopathy (DR). These complications arise from hyperglycemia and may lead to reduced life expectancy and disability [2]. For example, it is estimated that 1 in 14 people have diabetes in the United Kingdom, and it is one of the leading causes of sight loss in working-age adults [3,4]. In addition, patients with diabetes are known to present late to clinics and have a high missed appointment rate (>10%), which regularly results in avoidable deterioration of patients’ quality of life [5,6].

Digital health technologies (DHTs) often target these clinically complex patients to prevent the worsening of diabetic conditions through self-monitoring. Digital health apps, ranging from mobile apps that track dietary intake and blood glucose levels to systems that provide reminders for health appointments, are at the forefront of a shift toward more personalized and proactive health care [7,8]. Additionally, research suggests that app use increases the management of diabetes by enabling better self-care, improving glycemic control, and prompting early intervention and deterioration to prevent the onset of diabetes-related complications [9,10]. However, the adoption of these technologies is hindered by a myriad of factors, including but not limited to user accessibility challenges, limited usability, and an insufficient consideration of those with vision or hearing impairments [8,11]. Apps are designed and developed differently and may miss crucial aspects of development, due to a lack of clinical expertise, a lack of iterative user feedback, fast development, and limited research funding [12].

Recent research suggests that most diabetes digital health apps remain largely unregulated [13]. Across the literature, no universally accepted standard for evaluating app development currently exists, and multiple systematic and scoping reviews have called for clearer guidance to improve the quality, efficacy, safety, content, and consistency of digital health apps [14-17]. Several recommendations have emerged to guide future research, including the development of guidelines that integrate behavior change theory, incorporate iterative user feedback, improve privacy and data security, and enhance accessibility and data sharing with health care professionals [13,18].

To address these shortcomings and improve the standardization of the app development pipeline, the National Health Service Digital (NHSX) created the Digital Technology Assessment Criteria (DTAC) [19]. This framework aims to help give patients and professionals confidence that the apps they adopt meet minimum standards of interoperability, security, data protection, safety, accessibility, and usability standards [19]. The criteria should also act as a standards framework for future developers. The framework applies to all diabetes digital health apps, from apps used by the public to those used within hospitals, and contain criteria relevant to the development of apps internationally. The criteria aim to promote the consistent design and development of digital technology and cover five core areas, including the quality and safety of medical devices, and ensure user agreements and moderation are in place so that users are protected [19]. In addition, the assessment of interoperability ensures the data provided are communicated accurately, and usability and accessibility testing ensure that diverse populations can use them.

Complementing DTAC, the National Institute for Health and Care Excellence (NICE) released the Evidence Standards Framework [20], which contains standards used to develop a wide variety of DHTs. These standards were created from iterative input from thought leaders, stakeholders, developers, and system partners. The guidelines can be used to identify and evaluate new health apps and to conduct ongoing app evaluations. The NICE framework [20] provides a set of standards that developers can use to understand what compliant apps look like and how to demonstrate the effectiveness and value of health apps to investors. There are several categories that apps need to adhere to in order to show the apps are compliant. These include effectiveness of treatment; monitoring; calculate or diagnose functions; use of appropriate behavior change techniques; reliable information content; and ongoing data to show usage and value, in addition to quality and safeguarding, acceptability with users, equality considerations, and accurate and reliable measurements [20]. Together, the DTAC and NICE digital health guidelines (DHG) form a comprehensive and structured model for digital health app design, development, and evaluation.

Although DTAC [19] and the NICE DHG [20] have set forth standards aimed at ensuring the safety and usability of health apps, a disconnect remains between these theoretical guidelines and the practical realities of app development, where rapid market-driven development cycles often overshadow the meticulous process of iterative testing and user feedback integration. This gap not only undermines the potential benefits of digital health solutions but also raises concerns about user safety, data privacy, and the exacerbation of existing health inequalities. A gap also exists in the literature on whether diabetes digital health apps consistently adhere to such standards or any at all. Previous reviews have primarily focused on individual elements of app development, such as clinical effectiveness [21,22], usability features [13], and accessibility challenges [15,18]. However, few have evaluated app development against established digital health frameworks.

This systematic review aimed to bridge this gap by identifying relevant diabetes digital health apps and critically evaluating how their design process performs against the best-practice evidence standards frameworks for DHTs [19,20]. By doing so, it sought to underscore the value of a structured, evidence-based approach to the development of DHTs, one that goes beyond mere functionality to ensure comprehensive safety and equity in diabetes management.

## Methods

### Study Design

A systematic literature review was undertaken on published studies reporting the design and development of digital tools to support populations with diabetes. The methodology adopted for the systematic review followed the University of York Centre for Reviews and Dissemination [23], the *Cochrane Handbook on Systematic Reviews of Interventions* [24], and PRISMA (Preferred Reporting Items for Systematic Reviews and Meta-Analyses) guidelines [25]. The review was registered on PROSPERO in April 2022 (CRD42022322040) and conducted according to our pre-established protocol. It was reported using PRISMA guidelines (Multimedia Appendix 1) [26].

### Search Strategy

We conducted a systematic review of studies on diabetes digital health apps to report their adherence to design and development guidelines [19,20]. We analyzed 6 years (January 2017-November 2023) of diabetes digital health app development papers published in English. We searched the MEDLINE and Embase databases using the strategy outlined in Table 1, with a combination of free text and Medical Subject Headings (MeSH) for the search terms. Embase and MEDLINE were used as they have the highest two-database recall rate (92.8%) compared to other databases [27]. The key terms we identified were combined using the Ovid platform’s operators “AND/OR.” Reference lists of the included papers were also searched, authors of key papers were identified, and their publication lists were checked for other relevant research. The searches were also rerun just before the final analyses to identify and retrieve any further studies for inclusion.

**Table 1:** Search strategy used in MEDLINE and Embase to identify eligible studies on diabetes digital health apps for inclusion.

Database	Search strategy	Number of references
MEDLINE	1. Smartphone/ 2. Mhealth.mp. 3. mobile applications/ 4. digital intervention*.mp. 5. exp Diabetes Mellitus/ 6. user-centered design/ 7. Design.mp. 8. Adherence.mp. 9. 1 or 2 or 3 or 4 10. 6 or 7 or 8 11. 5 and 9 and 10 12. limit 11 to (English language and humans and yr=“2017-Current”)	258
Embase	1. Smartphone/ 2. Mhealth.mp. 3. mobile application/ 4. digital intervention*.mp. 5. exp diabetes mellitus/ 6. user-centered design/ 7. Design/ 8. Adherence.mp. 9. 1 or 2 or 3 or 4 10. 6 or 7 or 8 11. 5 and 9 and 10 12. limit 11 to (human and English language and yr=“2017-Current”)	322

The citations and abstracts of the search results were imported into Covidence systematic review software [28], and duplicates were removed. The records identified in the search strategy were downloaded and uploaded to Covidence. When screening the studies, we included those on the design or development of digital health apps used exclusively by patients with diabetes that were available on a smartphone or tablet. There were no restrictions on the type of diabetes or the age of the participants. We excluded studies on diabetes health technologies not available on a smartphone or tablet, papers solely providing opinions, industry reports, studies on practitioner behaviors, and reports of individual users or cases. Table 2 lists the inclusion and exclusion criteria used. Eligibility was determined based on paper type, language, population, intervention, outcomes, and publication date. Studies were included if they reported on the use, design, or development of mobile apps.

**Table 2:** Inclusion and exclusion criteria used to select studies for a systematic review of digital health apps for diabetes.

Criterion	Inclusion criteria	Exclusion criteria
Paper type	Peer-reviewed original research papers	Editorials, commentaries, opinion pieces, conference abstracts, reviews
Language	English	Papers published in languages other than English
Population	Adults with diabetes using a mobile health app	Studies focusing exclusively on clinicians or populations without diabetes
Intervention	Mobile apps designed specifically for diabetes self-management or monitoring	Nondigital interventions, web-only platforms, general health apps not specific to diabetes
Outcomes	Studies reporting quality features (eg, usability, accessibility, validation)	Studies lacking user-related outcomes or app quality assessment
Publication date	Studies published between 2017 and 2023	Studies published before 2017

Two reviewers (authors SLW and HM) independently screened the titles and abstracts of the selected studies for inclusion by applying eligibility criteria and selecting studies for inclusion in the systematic review. Any discrepancies in the findings were resolved through discussion with the third senior reviewer (author REH). One reviewer extracted relevant data from the included studies, and 10% were checked for accuracy by a second reviewer. Reviewer decisions and the reasons studies were excluded were recorded. A framework was created based specifically on the items included in DTAC [19] and the NICE DHG [20], where studies were scored on adherence (Table 3); each category was evaluated using a yes/no scoring system. The second reviewer checked 10% of the guideline scores against the full texts. The primary outcome of the systematic review was framework adherence scores for each diabetes digital health app included in the study using DTAC [19] and the NICE DHG [20]. These scores were presented as summary statistics.

The data extracted from each study included the following:

- Details of the study: country of origin, number of participants, participants’ baseline characteristics and demographics, details and features of the DHT, and year the research was conducted
- Methodology: details of the app design process, study design, type of diabetes classification, length of the study, and duration of user testing
- Outcome data: recruitment information from the validation studies, including the number of users who did not complete the research; main outcomes; user feedback; app accuracy; and usability analysis

**Table 3:** Scoring framework developed to assess adherence of studies on diabetes digital health apps to 13 binary criteria derived from DTAC^a^ 2021 and NICE^b^ 2022 development guidelines.

Binary criterion	Adherence assessment
Quality and safeguarding	The paper will score a “yes” for this category if it demonstrates any safeguarding measures in place to ensure the safety of vulnerable users and in peer-to-peer communication (eg, through user agreements or moderation). The developers must describe who has access to the platform, what their roles are within the platform, and why these people or groups are suitable and qualified to have access.
Acceptability with users	If representatives from intended user groups were involved in the design, development, or testing of the DHT^c^, the paper will score a “yes.” Depending on who is intended to operate the DHT, the intended users may include patient groups and service users, or health and care professionals. The developers must describe how user acceptability was appraised and provide any available data to show user acceptability with the DHT.
Equalities considerations	The paper will score a “yes” if it demonstrates that health inequality considerations have been factored into the design of the DHT. The developers must describe how this has been approached and included in the design. They must also describe any specific positive impacts and any efforts to reduce negative impacts on health inequalities. Evidence, if relevant, must also be provided that the DHT challenges health inequalities in the UK health and social care system; improves access to care among hard-to-reach populations; or promotes equality, eliminates unlawful discrimination, and fosters good relations between people with protected characteristics.
Accurate and reliable measurements	If there is available evidence that the data generated or recorded by the DHT are accurate, reproducible, and relevant to the range of values expected in the target population, the paper will score a “yes”. In addition, data will be accepted that show that the DHT is able to detect clinically relevant changes or responses.
App accuracy	The paper will only score a “yes” if it is appropriately tested and fit for purpose. Some types of accepted accuracy testing include: Validation testing: that the product design serves the intended purpose). This can include end-user testing and acceptance. Verification testing (functional correctness): Checking that the requirements of the product have been appropriately implemented. Load testing: that it performs reliably under continued stress and load. Performance testing: that it maintains responsiveness under various loading conditions. Regression testing: to prove that the product still performs as expected following a change or update.
App design process: demonstrating effectiveness for preventative behavior change or self-manage functions	The paper will score a “yes” if high-quality observational or quasi-experimental studies demonstrate relevant outcomes. These studies should present comparative data on relevant outcomes in a control group, the use of historical controls, and routinely collected data. Relevant outcomes may include behavioral or condition-related user outcomes, such as an improvement in condition management; evidence of positive behavior change; user satisfaction; patient-reported outcomes (preferably using validated tools), including symptom severity or quality of life or other clinical measures of disease severity or disability.
Demonstrating effectiveness for treatment, active monitoring, or calculate or diagnose functions	To score a “yes,” the paper must demonstrate the effectiveness of the DHT’s ability to treat, monitor, calculate, or diagnose a specific condition. One or more high-quality interventional studies (experimental or quasi-experimental design) to support the claimed benefits of the DHTs in a setting relevant to the UK health and social care system and showing improvements in relevant outcomes would be suitable. Examples include clinically relevant outcomes, patient-relevant outcomes, diagnostic accuracy, patient-reported outcomes, other clinical measures of disease severity or disability, healthy behaviors, physiological measures, user satisfaction, and user engagement.
Use of appropriate behavior change techniques	The paper will score a “yes” if it demonstrates evidence to support key factors, such as the choice of behavior change techniques used in the DHT. The DHT must provide personalized information or guidance using behavior change techniques to promote good health and healthy lifestyles. It must be consistent with recognized behavior change theory and recommended practice (aligned to guidance from NICE or relevant professional organizations) and appropriate for the target population.
Reliable information content	If a paper demonstrates valid health information, text, video, or other educational material for people, patients, or health care professionals to help them better understand their health and care, it will be scored a “yes.” This could include information about conditions, tests, or treatments. The developer should be able to show that processes are in place to maintain any health information provided by the DHT, which are valid (aligned to the best-available sources, such as the NICE DHG^d^, relevant professional organizations, or recognized UK patient organizations, and appropriate for the target population); accurate, reviewed, and updated by relevant experts (eg, health and care professionals in the relevant field) at defined intervals, such as every year; and sufficiently comprehensive.
Ongoing data collection to show usage of the DHT	The paper will score a “yes” if it demonstrates a plan for ongoing data collection to report the ongoing usage of the DHT in the target population in line with the expected usage profile or anonymized, aggregate data to show service user outcomes or any other outcomes collected by the DHT.
Ongoing data collection to show value of the DHT	If a commitment to ongoing data is collected on user outcomes or user satisfaction to show ongoing value, the paper will score a “yes.” The evidence should show that using the DHT impacts clinical management of the relevant condition in a setting relevant to the UK health and social care system or that it provides reliable test results that can be used to impact clinical management. Outcomes relevant to the intended purpose (value proposition) and claimed benefits of the DHT should be captured.
User feedback	If the developers can demonstrate how they have designed and evaluated their product with users during every stage of the life cycle, the paper will score a “yes.”
Usability and acceptability	The paper will score a “yes” if it demonstrates that the technology is easy to use and accessible to all users. It matters that the technology is not solely for the digitally literate, as this could disadvantage important groups of people.

^a^DTAC: Digital Technology Assessment Criteria.

^b^NICE: National Institute for Health and Care Excellence.

^c^DHT: digital health technology.

^d^DHG: digital health guidelines.

### Ethical Considerations

Ethical approval was not required as this study was a systematic review.

## Results

### Study Selection

**Figure 1:**
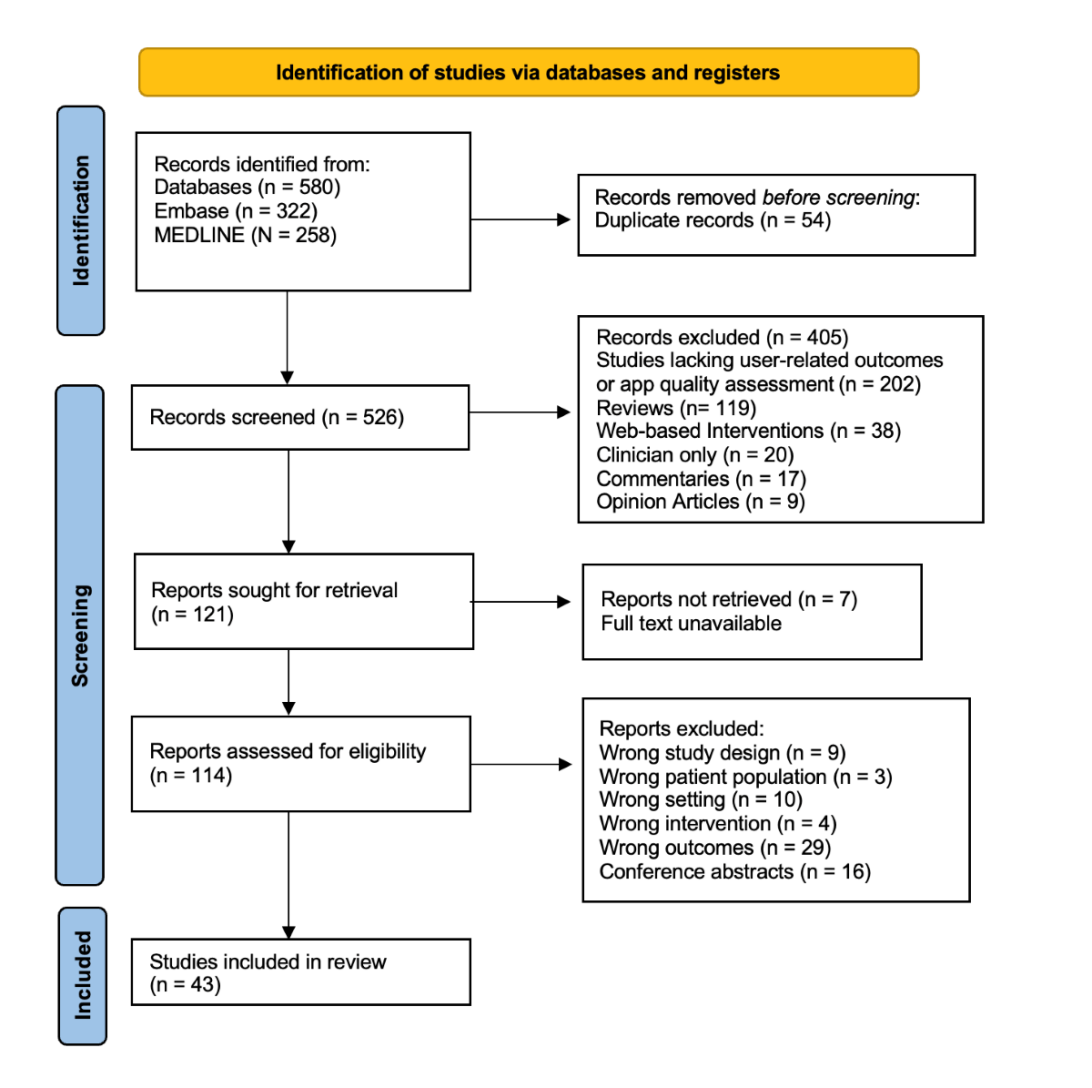
PRISMA flowchart for study selection, illustrating the identification, screening, and selection process for included studies. The figure shows the number of records retrieved from MEDLINE and Embase, the reasons for exclusion, and the final number of studies included in the review. PRISMA: Preferred Reporting Items for Systematic Reviews and Meta-Analyses.

The initial search from January 2017 to November 2023 identified 580 studies from MEDLINE and Embase. At this early stage, 54 (9.3%) duplicates were removed, and of the remaining 526 (90.7%) papers, 405 (69.8%) were excluded as they did not meet the inclusion criteria. These studies were removed as they lacked user-related outcomes and app quality assessment (n=202, 49.9%), focused on web-based interventions (n=38, 9.6%), or were reviews (n=119, 29.4%). Subsequently, 121 (23%) papers were retrieved, of which 7 (5.8%) were removed as the full text was not available for download. This left 114 (94.2%) full-text papers to be reviewed for eligibility. Of these, 43 (37.7%) met the inclusion criteria [29-71], and 71 (62.3%) were excluded due to being conference abstracts (n=16, 22.5%), having inappropriate outcomes (n=29, 40.8%), having an incorrect study design (n=9, 12.7%), and having an inappropriate setting (n=10, 14.1%). Figure 1 depicts the flowchart of study selection.

### Data Analysis

Data extracted (Multimedia Appendix 2) showed that the 43 studies [29-71] spanned a range of populations with diabetes, including type 1 diabetes (n=10, 23.3%), type 2 diabetes (n=22, 51.2%), both type 1 and type 2 diabetes (n=5, 11.6%), gestational diabetes (n=5, 11.6%), and diabetes and hemoglobin A_1c_ (HbA_1c_)>6.5% (n=1, 2.3%). The studies also explored a range of diabetes health-related functions (eg, self-management, medication adherence, lifestyle changes, and diabetes education) [29-71].

The methodology data extracted from the included studies identified the details of the app design process, study design, type of diabetes classification, length of the study, duration of user testing, and items from DTAC [19] and NICE [20] guidelines. The items used from DTAC [19] and NICE [20] guidelines are shown in Table 4 and Multimedia Appendix 3. Results of the review are presented using descriptive statistics.

**Table 4:** Adherence results of 43 studies included in a systematic review of diabetes digital health apps, indicating how many studies met the DTAC^a^ 2021 and NICE^b^ 2022 criteria.

Criterion	Score=no, n (%)	Score=yes, n (%)
Quality and safeguarding	34 (79.1)	9 (20.9)
Acceptability with users	23 (53.5)	20 (46.5)
Equality considerations	36 (83.7)	7 (16.3)
Accurate and reliable measurements	38 (88.4)	5 (11.6)
App accuracy	37 (86.0)	6 (14.0)
App design process: demonstrating effectiveness for preventative behavior change or self-management functions	25 (58.1)	18 (41.9)
Demonstrating effectiveness for treatment, active monitoring, or calculate or diagnose functions	25 (58.1)	18 (41.9)
Use of appropriate behavior change techniques	20 (46.5)	23 (53.5)
Reliable information content	30 (69.8)	13 (30.2)
Ongoing data collection to show usage of the DHT^c^	29 (69.0)	14 (31.0)
Ongoing data collection to show value of the DHT	26 (60.5)	17 (39.5)
User feedback	5 (11.6)	38 (88.4)
Usability and acceptability	12 (27.9)	31 (72.1)

^a^DTAC: Digital Technology Assessment Criteria.

^b^NICE: National Institute for Health and Care Excellence.

^c^DHT: digital health technology.

Based on the recommended design and development criteria, 40 (93%) of the 43 studies received 6 “yes” scores or lower; only 3 (7%) studies scored between 7 and 9. No studies adhered to all the DTAC [19] and NICE [20] minimum development standards. A high percentage of the studies (n=36, 83.7%) did not adhere to guidelines on equality considerations, provide sufficient evidence of accurate and reliable measurements (n=38, 88.4%), assess app accuracy (n=37, 86%), or provide appropriate quality and safeguarding (n=34, 79.1%).

## Discussion

### Principal Findings

In critically examining the adherence of diabetes DHTs to DTAC [19] and the NICE DHG [20] frameworks, our review highlights a large gap between theoretical ideals and the practical realities of app design, development, and evaluation. Despite the well-intentioned criteria of these frameworks to establish standards for safety, usability, and efficacy, there are significant differences in how developers design and test diabetes health technology. Not a single study met the selected minimum development standards, as outlined, and 93% failed to meet even 40% of these standards, which suggests that researchers and developers focus on app development to the detriment of comprehensive evaluation and adherence to these quality standards. This oversight not only raises questions about how developers perceive quality apps but also queries whether this is a systemic issue within the diabetes DHT sector.

This lack of alignment may be attributed to the overwhelming range of frameworks assessing app quality, leading to confusion among developers about what constitutes a quality app. The criteria of frameworks that assess app quality also vary substantially, making it difficult for developers to know what a quality app needs. For example, a systematic review showed that in 2018, a total of 48 different international frameworks with different criteria were in circulation to rate the quality and usability of digital health apps [72]. A common theme between these frameworks is the inclusion of user feedback and usability in both general health apps [73] and diabetes-specific health apps [74]. This still seems to be the case, and it had a knock-on impact on developers’ focus in this review, with user feedback (88.4%) and usability and acceptability (72.1%) scoring the highest. It seems usability has often been foregrounded at the expense of comprehensive efficacy testing, as developers seem to interpret a quality app as one that gathers user feedback and usability over strict adherence to quality, accuracy, and safety guidelines.

Although usability is undeniably important, the emphasis on iterative user feedback—a practice not uniformly adopted across the 43 studies—underscores the need for a more systematic and inclusive approach to app development. People with diabetes tend to have other comorbid conditions or impairments, making the accessibility and usability of DHTs a priority [75]. Ensuring that the needs of these users are understood and met is central to the design process, and poorly designed apps create barriers that increase health inequalities and prevent the uptake and long-term usage of apps [76]. However, it simply is not enough to gain user feedback once. Prior to the rapid software deployment of the apps, user input should take the form of consultation, testing, and feedback throughout development [77,78]. Regular iterations and collaborations with users within populations with diabetes are needed to pinpoint functionalities that may be unsuitable [79].

The amount of iterative user and clinician feedback incorporated in the process highly varied in our included studies [29-71] and, within existing research, ranged from 0% to 87% [80]. Similar findings were also present in research by Fu et al [81], whose analysis of several usability studies reported poor (38%) to average (80%) rates of user feedback. This iterative user feedback goes hand in hand with data on adherence, where users help create apps they are willing to use. Fu et al [81] also found that apps with design flaws (eg, Manual blood sugar level entry) increase user error and result in limited adherence and more varied user input.

If research suggests that poorly developed apps result in less adherence, it seems clear why developers should adhere to these frameworks but not why they do not [81]. One explanation is that the role of health care professionals in endorsing apps for monitoring, diagnosing, treating, and calculating further complicates things. In the United Kingdom, for example, research suggests that 77% of National Health Service (NHS)–recommended high-risk digital health apps do not have any peer-reviewed evidence [82]. This was the case for many categories, including app effectiveness, accuracy, usability, and acceptability. This is particularly striking as health care professional approval could impact whether apps are recommended by clinicians and used by patients [83]. Developers are permitted to reference expert opinion without explicit critical evaluation, or based on physiology, bench research, or first principles [84]. Additionally, there is no requirement for the evidence to undergo peer review or to be accessible to the public. It seems that if developers do not need to provide evidence before their app can be used, they will not do it, which underscores the need for a more rigorous and transparent evaluation process. Another explanation for the lack of adherence may be the complexity and time commitment of evaluating apps. Funding for these apps tends to come alongside tight turnaround times and pressure to meet grant and investor deadlines [85]. Therefore, some start-ups and established companies may prioritize a working prototype rather than the accuracy of content provided in their apps [85]. This fast development time means that only a small number of apps show, involve, and receive feedback from medical professionals during app development [86].

In addition, user, content, platform, and interface components are continually evolving. They can be updated, modified, released, or removed by developers, meaning that the collected feedback may no longer be relevant. Additionally, to keep up with these continually evolving apps, frameworks are updated, leaving developers “chasing their tail” to keep up with the latest requirements.

Ultimately, this lack of adherence has led to a concerning oversight of equality considerations, accurate measurements, and user safety. Across our included studies, a high percentage did not adhere to guidelines on equality considerations (83.7%), provide sufficient evidence of accurate and reliable measurements (88.4%), assess app accuracy (86%), or provide appropriate quality and safeguarding (79.1%). While acknowledging the strengths of this review, particularly its exploration of adherence in diabetes apps to design and development guidelines, [73] it is also necessary to recognize its limitations. Moreover, privacy and safeguarding seem to be a reoccurring concern across the field, where poorly secured personal health information and inaccurate apps have the potential to impact patient safety [87-89]. This was also found by Sharma et al [74] in diabetes management, where equality considerations and privacy and safeguarding scored 50% and 40%, respectively. Such oversights not only compromise the safety and efficacy of diabetes digital health apps but also challenge health care providers and users in identifying trustworthy apps. Our review suggests a pressing need for a unified approach toward adherence to quality and safety guidelines, emphasizing the importance of integrating user concerns early in the development process to enhance reliability, security, and inclusivity.

A fundamental way to improve these scores would be to adhere to the key criteria from DTAC [19] and the NICE DHG [20] more stringently, while taking user concerns on board during development or before it entirely.

### Strengths and Limitations

The exploration of adherence of diabetes digital health apps to design and development guidelines [19,20] was a strength of this review. While acknowledging the strengths of this review, it is also necessary to recognize its limitations.

None of the apps were downloaded, as some apps required a unique patient access code or were restricted to clinical trials. The review relied on app descriptions for the scoring of studies, which may not fully capture the nuanced and dynamic nature of app development and user interaction. This approach was used to assess apps based on the developers’ stated intentions in the peer-reviewed literature. Although downloading an app may offer insight into some app features (ie, surface-level accessibility and usability), it provides limited information about the underlying design rationale, validation evidence, and other criteria assessed in this review (eg, clinical effectiveness, preventative behavior outcomes, accuracy and reliability of measurements, safeguarding features, and integration of user feedback) [90]. Therefore, although there is some value to downloading the apps, it would not have meaningfully enhanced the quality or depth of our assessment, particularly given that many of the criteria we evaluated were not readily observable through the app interface alone. For this reason, the absence of app downloads should not compromise the rigor or relevance of our review.

Moreover, the use of an unvalidated measurement tool that takes individual items from separate frameworks to assess app quality and usability may have introduced a degree of subjectivity and potential bias into the analysis. However, it offered greater flexibility than the frameworks individually for this analysis. A formal checklist developed through consensus with relevant stakeholders and experts that encompasses all these items, akin to the CONSORT (Consolidated Standards of Reporting Trials) checklist for clinical trials [91], the Evidence DEFINED (Evidence in Digital Health for Effectiveness of Interventions With Evaluative Depth) framework for intervention studies [92], or the STARD (Standards for Reporting of Diagnostic Accuracy) statement for diagnostic test accuracy studies [93], would be an ambition that can be used by regulatory bodies and journals to assess a new app’s adherence to best-practice guidelines.

### Recommendations

Currently, the multiplicity of international frameworks creates additional confusion for developers, particularly those attempting to adhere to guidelines across multiple countries. To address this point, we suggest a set of core *essential quality criteria* that appear consistently across international frameworks and are particularly critical for diabetes digital health app development: safety, accurate and reliable information, usability, data privacy, and clinical effectiveness. These domains appear across the United Kingdom’s Medicines and Healthcare Products Regulatory Agency (MHRA) guidelines [94], the European Union’s Medical Device Regulation (MDR) [95], and digital health guidelines from the US Food and Drug Administration (FDA) [96], Brazil’s Agência Nacional de Vigilância Sanitária (ANVISA) [97], Health Canada [98], China’s National Medical Products Administration (NMPA) [99], and Japan’s Pharmaceuticals and Medical Devices Agency (PMDA) [100]. By foregrounding these shared principles, developers may be able to design applications with a *universal quality baseline* that aligns with global expectations, even if the ambition for full compliance with every national framework remains challenging.

Nonetheless, certain country-specific requirements, particularly those related to device regulations, data security, and privacy, are unlikely to be harmonized in the future. For example, developers targeting the United Kingdom must comply with the UK Medical Device Regulations (UK MDR) of 2002 and the Medicines and Medical Devices Act of 2021, the United States mandates FDA compliance [96], and other jurisdictions (eg, Brazil’s ANVISA [97], Japan’s PMDA [99], or China’s NMPA [100]) impose unique regulatory expectations each. As such, a fully unified framework is currently impractical, particularly for developers seeking regulatory clearance in multiple regions.

To address this, we propose that developers adopt a tiered compliance strategy: designing digital health apps to align with shared international principles (eg, usability, safety, and efficacy), while preparing supplemental documentation and adjustments to meet specific regulatory requirements of each target country. Furthermore, ensuring transparency in reporting the regulatory status of digital health tools and publishing postmarket surveillance data, where available, may help bridge current gaps in defining and recognizing quality internationally.

### Conclusion

In conclusion, our results demonstrate that most diabetes digital health apps do not abide by best-practice development standards. The findings call for a collaborative effort to align app development more closely with the criteria identified in DTAC [19] and NICE [20] guidelines, ensuring that DHTs not only meet the needs of users but also do so in a manner that is safe, effective, and equitable. This endeavor is not just about improving the technological aspects of health apps but also about rethinking the entire ecosystem in which they are created, evaluated, and used to prevent research wastage in the longer term.

## Appendix

Multimedia Appendix 1 PRISMA checklist.

Multimedia Appendix 2 Details of the 43 studies.

Multimedia Appendix 3 Bar chart showing the percentage of included studies (N=43) that met each of the 13 evaluated development criteria, grouped by domain (ie, user engagement, behavior change, monitoring), as derived from the DTAC 2021 and NICE 2022 digital health frameworks. DTAC: Digital Technology Assessment Criteria; NICE: National Institute for Health and Care Excellence.

## Abbreviations

ANVISA: Agência Nacional de Vigilância Sanitária
CONSORT: Consolidated Standards of Reporting Trials
DF: diabetic foot
DHG: digital health guidelines
DHT: digital health technology
DN: diabetic nephropathy
DR: diabetic retinopathy
DTAC: Digital Technology Assessment Criteria
Evidence DEFINED: Evidence in Digital Health for Effectiveness of Interventions With Evaluative Depth
FDA: Food and Drug Administration
HbA1c: hemoglobin A1c
MDR: Medical Device Regulation
MeSH: Medical Subject Headings
MHRA: Medicines and Healthcare Products Regulatory Agency
NHS: National Health Service
NHSX: National Health Service Digital
NICE: National Institute for Health and Care Excellence
NMPA: National Medical Products Administration
PMDA: Pharmaceuticals and Medical Devices Agency
PRISMA: Preferred Reporting Items for Systematic Reviews and Meta-Analyses
STARD: Standards for Reporting of Diagnostic Accuracy
UK MDR: UK Medical Device Regulations

## Data Availability

The datasets analyzed in this study are not currently publicly available, as they are intended for inclusion in an upcoming doctoral thesis submission. The data will be made openly accessible following formal submission and archiving through the Queen’s University Belfast institutional repository. Until then, data may be made available upon reasonable request to the corresponding author in accordance with institutional data-sharing policies.
