# Supplementary material for: Adherence to Usability and Accessibility Principles in Digital Health Applications for Patients With Diabetes: Systematic Review
Source: J Med Internet Res. 2025 Sep 26;27:e71567. doi: 10.2196/71567 (PMC12514418; doi:10.2196/71567)
Supplement: Multimedia Appendix 2 [file jmir_v27i1e71567_app2.docx]

**Multimedia Appendix 2.** Details of the 43 studies: country of origin, number of participants, participant baseline characteristics, demographics, details and features of the digital health technology, and year the research was conducted/published.

| Study title | Author name | Year the research was conducted/ published | Type and features of the digital health technology | Participant baseline characteristics | Number of participants | Country of origin |
| --- | --- | --- | --- | --- | --- | --- |
| The development of My Care Hub mobile-phone app to support self-management in Australians with type 1 or type 2 diabetes | Adu et al [1] | 2020 | A tool for monitoring self-management activities, providing access to information and aiding motivation to engage with diabetes self-management. | Type 1 and type 2 diabetes | 12 (8 without diabetes and 4 with diabetes) | Australia |
| Caregiver's opinions on the design of the screens of a future gamified mobile application for self-management of type 1 diabetes in children in Saudi Arabia | Alsalman et al [2] | 2021 | gamified mobile application designed for the self-management of type 1 diabetes in children. It incorporates features such as caring for a character, challenging friends, points, level, leaderboard as rewarding principles, reminders, and notifications for doctor's appointments, insulin injection times, blood glucose readings, and tips for medication adherence, increasing blood glucose readings, supporting physical activities, and healthy eating habits. | Type 1 diabetes | 65 | Saudi Arabia |
| Development and validation of a MHEALTH technology for the promotion of self-care for adolescents with diabetes | Alves et al [3] | 2021 | The research aimed to develop and validate a mobile application (APP) to promote self-care for adolescents with DM1. | Type 1 diabetes | 8 | Brazil |
| Usability of an intelligent virtual assistant for promoting behavior change and self-care in older people with type 2 diabetes | Balsa et al [4] | 2020 | Supporting older people with Type 2 Diabetes Mellitus (T2D) in medication adherence and lifestyle changes. The virtual assistant, named Vitória, is an anthropomorphic character capable of articulating speech, expressing emotions through facial and body animations, and engaging in intelligent relational interactions with users. | Type 2 Diabetes | 20 | Portugal |
| Use of brief messages based on behavior change techniques to encourage medication adherence in people with type 2 diabetes: developmental studies | Bartlett et al [5] | 2020 | To develop in app messages that have proven fidelity to specified evidence-derived behavior change techniques in people with T2D. | Type 2 Diabetes | 123 | United Kingdom |
| Development and assessment of a mobile health application for monitoring the linkage among treatment factors of type 1 diabetes mellitus | Bellei et al [6] | 2020 | To present Soins DM, a mobile health tool, for monitoring the linkage among treatment factors of T1DM with an interactive data visualization approach. | Type 1 diabetes | Prototype questionnaire testing:76, Pilot app testing: 110 | Brazil |
| Web-based intervention for women with type 1 diabetes in pregnancy and early motherhood: critical analysis of adherence to technological elements and study design | Berg et al [7] | 2018 | To critically analyze and discuss the challenges of conducting a Web-based health intervention as a randomized controlled trial. | Type 1 diabetes | 83 using MODIAB Web-based support | Sweden |
| How mHealth can facilitate collaboration in diabetes care: qualitative analysis of co-design workshops | Bradway et al [8] | 2020 | To create a mHealth data-sharing system for diabetes, to present patient-gathered mHealth data to be used effectively by both parties to facilitate shared-decision making and collaboration in diabetes care. | Type 1 and type 2 diabetes | 15 | Norway |
| Who needs what (features) when? Personalizing engagement with data-driven self-management to improve health equity | Burgermaster et al [9] | 2023 | Platano, designed for the self-management of type 2 diabetes (T2D). Platano includes a basic set of features for logging meals and blood glucose levels, which are crucial for individuals with T2D. The app allows users to log their meals by photographing them and providing a brief textual description, as well as entering their blood glucose levels from their glucometers. | Type 2 diabetes | 53 | USA |
| Implementing a text message–based intervention to support type 2 diabetes medication adherence in primary care: a qualitative study with general practice staff | Butler et al [10] | 2023 | It is a mobile phone-based intervention delivering brief messages targeting identified behavior change techniques promoting medication use to people with type 2 diabetes in general practice. -The SuMMiTD intervention aims to address limitations with existing text message-based interventions by taking a theory and evidence-based approach to intervention development, drawing on the perspectives and preferences of people with type 2 diabetes in refining message content, and clearly specifying the content of messages included in the intervention. -The proposed intervention contains over 300 unique messages based on established behavior change techniques and has demonstrated good acceptability among people with type 2 diabetes. -The intervention is designed to target a broad range of individuals with type 2 diabetes and to support people in both the initiation and implementation phases of medication adherence. | Type 2 | 46 | United Kingdom |
| Developing the "Healthcare CEO App" for patients with type 1 diabetes transitioning from adolescence to young adulthood: a mixed-methods study | Chiang et al [11] | 2023 | The app aims to address the specific care needs of adolescents with type 1 diabetes during the transition period, providing comprehensive functions and tailored content to meet their specific needs. The features of the digital health technology include health tracking, knowledge base, barrier-free communication, diet and exercise guidance, and a chat room for peer support. | Type 1 diabetes | 35 | Taiwan |
| Clinical outcomes of a digitally supported approach for self-management of type 2 diabetes mellitus | De Luca et al [12] | 2023 | DeLuca 2023 | Type 2 diabetes | 100 | Italy |
| User-centered development of a digitally-delivered dietary intervention for adults with type 2 diabetes: the T2Diet Study | Dening et al [13] | 2022 | To describe the user-centered approach used in the T2Diet Study to develop a new web-based dietary intervention for adults with T2D, exploring strategies for enhancing adherence and engagement. | Type 2 diabetes | 21 | Australia |
| User experience of an innovative mobile health program to assist in insulin dose adjustment: outcomes of a proof-of-concept trial | Ding et al [14] | 2018 | To develop an innovative mobile health (m-Health) mobile-based IDA program (mIDA) and evaluate the user adherence and experience through a proof-of-concept trial. | Type 2 diabetes | 9 | Australia |
| Using community health workers and a smartphone application to improve diabetes control in rural Guatemala | Duffy et al [15] | 2020 | To improve diabetes care in rural Guatemala through the development of a CHW-led diabetes program and a smartphone application to provide CHWW's with clinical decision support. | Type 2 diabetes | 89 | USA & Guatemala |
| Developing and testing an integrated patient mHealth and provider dashboard application system for type 2 diabetes management among Medicaid-enrolled pregnant individuals based on a user-centered approach: mixed-methods study | Fareed et al [16] | 2022 | Our objective is to describe a formative study that developed an integrated patient based mHealth and provider dashboard application system for management among Medicaid-enrolled pregnant patients with T2D. | Type 2 diabetes | 7 patients, 7 providers | USA |
| User-centered design to improve information exchange in diabetes care through eHealth: results from a small-scale exploratory study | Fico et al [17] | 2020 | To evaluate the use and acceptance of a self-management system for diabetes developed with User Centered Design Principles in community settings. | Type 1 and type 2 diabetes | 20 patients and 24 professionals | Spain and Germany |
| Design and testing of a smartphone application for real-time self-tracking diabetes self-management behaviors | Groat et al [18] | 2018 | iDECIDE, a smartphone application that gathers daily diabetes SMBs and CTs related to meal and alcohol intake and exercise in real-time, and contrast patients’ actual behaviors against those self-reported with the app. | Type 1 diabetes | Preliminary usability study: 5, 2nd 6, Pilot testing 14, | USA |
| The design and development of MyT1DHero: a mobile app for adolescents with type 1 diabetes and their parents | Holtz et al [20] | 2019 | MyT1DHero is an app that links parents and their children by creating two separate app interfaces, one for the parent and one for the child, that work together to help them communicate about diabetes management. | Type 1 diabetes | 10 adolescents, 10 parents | USA |
| A nudge-inspired AI-driven health platform for self-management of diabetes | Joachim et al [21] | 2022 | An online team-based game delivering diabetes self-management education (DSME) to patients via e-mail or mobile application (app) | Type 2 Diabetes | 213 | Australia |
| A team-based online game improves blood glucose control in veterans with type 2 diabetes: a randomized controlled trial | Kerfoot et al [22] | 2017 | An online team-based game delivering diabetes self-management education (DSME) to patients via e-mail or mobile application (app) | Type 2 Diabetes | 456 | USA |
| Developing and evaluating a mobile foot care application for persons with diabetes mellitus: a randomized pilot study | Kilic and Karadag [22] | 2020 | Using m-DAKBAS increases the knowledge level of patients with diabetes related to foot care, encourages patients with diabetes to form positive behaviors and increases the self-efficacy levels related to foot care. | Type 1 and type 2 diabetes | 10 | Turkey |
| User-centred development of an mHealth app for youth with type 1 diabetes: the challenge of operationalizing desired features and feasibility of offering financial incentives | Krmpotic et al [23] | 2022 | The Canadian Diabetes Incentives and Technology (CanDIT) app was developed to help adolescents with type 1 diabetes. | Type 1 diabetes | 6 | Canada |
| Intervention development of a brief messaging intervention for a randomised controlled trial to improve diabetes treatment adherence in sub-Saharan Africa | Leon et al [24] | 2021 | An evidence- and theory-informed brief messaging intervention, to improve diabetes treatment adherence in sub-Saharan Africa. | Type 2 Diabetes | 89 patient focus groups, 56 phase 3, 10 phase 4 | Malawi & South Africa |
| Definition and development of a digital system for the empowerment and activation of type 1 diabetic patient | Merino-Barbancho et al [25] | 2021 | A mobile application implementing an educational intervention module for the empowerment and activation of type 1 diabetic patients supporting them in their daily self-control to avoid and delay long-term complications. | Type 1 diabetes | 15 Design users; End evaluation: 3 diabetic patients, 3 endocrinologists | Madrid |
| Health-e mums: evaluating a smartphone app design for diabetes prevention in women with previous gestational diabetes | O'Reilly and Laws [26] | 2019 | A pilot smartphone app developed from an evidence-based diabetes prevention program specifically for women with previous gestational diabetes mellitus. | Gestational diabetes | 26 | Australia & Ireland |
| Enhancing system acceptance through user-centred design: integrating patient generated wellness data | Pais et al [27] | 2021 | The digital health technology discussed in the document is a clinical ecosystem designed to integrate patient-generated wellness data, specifically targeting women with gestational diabetes mellitus (GDM). The system includes features such as extracting, downloading, and storing data about blood glucose readings, food intake, physical exercise, and insulin dosage from remote devices used by women with GDM, such as mobile devices and glucose meters | Gestational diabetes | The study involved a total of 15 participants comprising ten clinicians and five women with gestational diabetes mellitus (GDM) | New Zealand |
| A mobile app for the self-management of type 1 diabetes among adolescents: a randomized controlled trial | Goyal et al [28] | 2017 | BANT is an app aimed to assist adolescents with the self-management of type 1 diabetes. | Type 1 diabetes | 92 | Canada |
| Development and testing of a mobile application to support diabetes self-management for people with newly diagnosed type 2 diabetes: a design thinking case study | Petersen and Hempler [29] | 2017 | A multifunctional app was useful for people with newly diagnosed type 2 diabetes including diabetes activities after diagnosis, recording of health data, reflection games and goal setting, knowledge games and recording of psychological data such as sleep, fatigue, and well-being. | Type 2 Diabetes | 26 | Denmark |
| Novel Bluetooth-enabled tubeless insulin pump: a user experience design approach for a connected digital diabetes management platform | Pillalamarri et al [30] | 2018 | The Omnipod DASH™ Insulin Management System incorporates several user-requested features based on extensive user research and feedback. These include the personalization of PDM lock screen, Food database for bolus calculator, alarms, and notifications as well as the wireless integration with the Dexcom CGM. The purpose of the Omnipod DISPLAY App is to provide users with the ability to view continuous glucose monitoring (CGM) data on the same screen as Omnipod DASH data on their personal smartphone. This feature allows for seamless integration and access to critical health data, enhancing the user's ability to monitor and manage their diabetes effectively. Additionally, the app serves as a companion tool for caregivers, enabling them to track patient data and provide support as needed. | T1DM and gestational | 343 | USA |
| Self-monitoring diabetes-related foot ulcers with the MyFootCare app: a mixed methods study | Ploderer et al [31] | 2023 | The MyFootCare app, is designed for individuals with diabetes-related foot ulcers (DFUs) to self-monitor their healing progression. The app includes features such as a motivational image on the home screen to visualize a goal, a progress graph showing changes in DFU size over time, a star on the graph to visualize the goal to reach a 50% reduction within 4 weeks, a gallery to review all foot checks, and notifications to take foot selfies, which can be tailored under settings. | Type 2 Diabetes | 12 | Australia |
| Development of a computer-aided text message platform for user engagement with a digital diabetes prevention program: a case study | Rodriguez et al [32] | 2021 | To develop a personalized automatic message system (PAMS) to promote user engagement to the dDPP platform by sending messages on behalf of their primary care provider. | Type 1 and type 2 diabetes | 9 patients; 4 clinicians | USA |
| Mobile-based and cloud-based system for self-management of people with type 2 diabetes: development and usability evaluation | Salari et al [33] | 2021 | Cloud-based and mobile-based diabetes self-management app designed to help people with diabetes change their health behavior and enable remote monitoring by health care providers. | Type 2 Diabetes | 14 patients, 7 healthcare providers | Iran & Australia |
| User-centered ‘Swayam Diabetes’m-health application for self-care management of type 2 diabetes in urban home settings: the usability and utility testing of mobile application and perspectives | Sinha et al [34] | 2023 | "The m-health application was designed for self-care management of type 2 diabetes and keeping the patient at the core, the application was designed, human-centered focusing on comprehensive self-care management, which included, daily dairy, nutrition, food update, exercise, medicine intake, and nutrition informatics to support for improving self-care management adherence to self-care activities." | Type 2 Diabetes | 250 | India |
| Applying the behaviour change wheel to develop a smartphone application 'stay-active' to increase physical activity in women with gestational diabetes | Smith et al [35] | 2022 | Behaviorally informed smartphone application (Stay-Active) for women attending an NHS GDM clinic. | Gestational diabetes | 64 | UK |
| Protocol for a qualitative study exploring the perception of need, importance and acceptability of a digital diabetes prevention intervention for women with gestational diabetes mellitus during and after pregnancy in Malaysia (Explore-MYGODDESS) | Sobri et al [36] | 2021 | A digital technology to be used among women and their healthcare providers (HCPs) to support healthy behavior changes in women with GDM. | Gestational diabetes and Type 2 Diabetes | Phase 1: 60 DM, 40 HCP's; Phase 2: 30 DM,15 HCP's | Malaysia & U. K |
| Facilitators and barriers to chronic disease self-management and mobile health interventions for people living with diabetes and hypertension in Cambodia: qualitative study | Steinman et al [37] | 2020 | To develop a mobile-based messaging app to link MoPoTsyo’s database, PEs, pharmacies, clinics, and people living with diabetes and/or hypertension to improve adherence to evidence-based treatment guidelines. The study developed mHealth messages to address barriers to chronic disease management, such as reminders about medications, laboratory tests, and doctor’s consultations, education on self-management, and support for obstacles to disease management. | The study involved 70 participants, including 59 individuals living with type 2 diabetes and/or hypertension (referred to as patients) and 11 peer educators (PEs) from Cambodia. | 70 | Cambodia |
| StepAdd: a personalized mHealth intervention based on social cognitive theory to increase physical activity among type 2 diabetes patients | Sze et al [38] | 2023 | StepAdd is a personalized smartphone-based mHealth intervention designed to increase physical activity among type 2 diabetes patients. It is based on social cognitive theory (SCT) and aims to assist patients in achieving significant improvements in daily step count, HbA1c, BMI, FBG, HDL-C, and TG. The intervention involves the use of a smartphone application, StepAdd, which provides personalized feedback, goal recommendations, and coping strategies based on the patient's own targeted step count and pedometer-captured data | Type 2 Diabetes | 33 | Japan |
| Exploring the consequences of food insecurity and harnessing the power of peer navigation and mHealth to reduce food insecurity and cardiometabolic comorbidities among persons with HIV: protocol for development and implementation trial of weCare/Secure | Tanner et al [39] | 2022 | weCare/Secure is a novel bilingual peer navigation-mHealth food insecurity intervention designed to reduce cardiometabolic comorbidities among food-insecure persons with HIV (PWH) living in the southeastern United States. The intervention also includes guidance on shopping for healthy foods on a limited budget, preparing foods according to recommendations for reducing cardiometabolic disease risks, and supporting provider communication. | Type 2 Diabetes | 2045. The study involves a total of 1800 participants in Aim 1, which is a longitudinal cohort study. Additionally, aim 2 will include 200 participants, with 100 randomized to the intervention arm and 100 to the usual care arm. Aim 3 will involve a subset of Aim 2 participants, with a sample size of 45 interview participants. | USA |
| Co-design of a digital dietary intervention for adults at risk of type 2 diabetes | Tay et al [40] | 2021 | A digital intervention that has been co-designed to specifically address the needs of adults at risk of Type 2 diabetes (T2D). | Type 2 Diabetes | 20 end users and 4 experts | Australia |
| Simplifying and personalising health information with mobile apps: translating complex models into understandable visuals | Waaler et al [41] | 2023 | The app is designed to simplify and personalize the presentation of scientific health information. It utilizes a statistical model to translate complex health research findings into user-centered visualizations that are easy to understand. The app is designed to automatically adjust the presentation of information based on the user's profile, offering individually tailored feedback on health and lifestyle. It integrates user data to provide personalized suggestions for lifestyle changes and their estimated health effects. The app aims to allow users to compare the health impact of various lifestyle factors and make informed decisions about improving their health. | Participants with diabetes (HbA1c≥6.5%) | 33,064 | Norway |
| Patient and provider perspectives on a novel mobile health intervention for low-income pregnant women with gestational or type 2 diabetes mellitus | Yee et al [42] | 2021 | A mHealth intervention to support pregnant women with DM, with particular attention to low-income women. | Type 2 or gestational DM | 45 | USA |
| Development and evaluation of DiabeText, a personalized mHealth intervention to support medication adherence and lifestyle change behaviour in patients with type 2 diabetes in Spain: a mixed-methods phase II pragmatic randomized controlled clinical trial | Zamanillo-Campos et al [43] | 2023 | Diabetext is a multifaceted digital health intervention designed to prevent type 2 diabetes mellitus (T2DM) by supporting lifestyle changes in individuals at risk of T2DM. It was created to address the urgent public health problem of T2DM and its associated complications, which have significant impacts on individuals' quality of life, life expectancy, and healthcare costs. The text messaging intervention involves the development of a library of messages covering relevant topics such as motivation, nutrition, physical activity, and sedentary lifestyle, adapted for the pre-diabetes population. The messages are designed to be personalized and cover a variety of topics, with progression in complexity, intensity, frequency, and volume in the case of physical activity. | Type 2 Diabetes | 420 | Spain |

## References

1. Adu MD, Malabu UH, Malau-Aduli AEO, Malau-Aduli BS. The development of My Care Hub mobile-phone app to support self-management in Australians with type 1 or type 2 diabetes. *Sci Rep*. Jan 08, 2020;10(1):7. doi: 10.1038/s41598-019-56411-0.
2. Alsalman DM, Ali ZB, Alnosaier Z, Alotaibi N, Alanzi TM. Caregiver's opinions on the design of the screens of a future gamified mobile application for self-management of type 1 diabetes in children in Saudi Arabia. *Int J Telemed Appl*. 2021;2021:8822676. doi: 10.1155/2021/8822676.
3. Alves L, Maia MM, Araújo M, Damasceno MMC, Freitas R. Development and validation of a MHEALTH technology for the promotion of self-care for adolescents with diabetes. *Cien Saude Colet*. May 2021;26(5):1691-1700. doi: 10.1590/1413-81232021265.04602021.
4. Balsa J, Félix I, Cláudio AP, Carmo MB, Silva ICE, Guerreiro A, et al. Usability of an intelligent virtual assistant for promoting behavior change and self-care in older people with type 2 diabetes. *J Med Syst*. Jun 13, 2020;44(7):130. doi: 10.1007/s10916-020-01583-w.
5. Bartlett YK, Farmer A, Rea R, French DP. Use of brief messages based on behavior change techniques to encourage medication adherence in people with type 2 diabetes: developmental studies. *J Med Internet Res*. May 13, 2020;22(5):e15989. doi: 10.2196/15989.
6. Bellei EA, Biduski D, Lisboa HRK, De Marchi ACB. Development and assessment of a mobile health application for monitoring the linkage among treatment factors of type 1 diabetes mellitus. *Telemed J E Health*. Feb 2020;26(2):205-217. doi: 10.1089/tmj.2018.0329.
7. Berg M, Linden K, Adolfsson A, Sparud Lundin C, Ranerup A. Web-based intervention for women with type 1 diabetes in pregnancy and early motherhood: critical analysis of adherence to technological elements and study design. *J Med Internet Res*. May 02, 2018;20(5):e160. doi: 10.2196/jmir.9665.
8. Bradway M, Morris RL, Giordanengo A, Årsand E. How mHealth can facilitate collaboration in diabetes care: qualitative analysis of co-design workshops. *BMC Health Serv Res*. Nov 30, 2020;20(1):1104. doi: 10.1186/s12913-020-05955-3.
9. Burgermaster M, Desai PM, Heitkemper EM, Juul F, Mitchell EG, Turchioe M, et al. Who needs what (features) when? Personalizing engagement with data-driven self-management to improve health equity. *J Biomed Inform*. Aug 2023;144:104419. doi: 10.1016/j.jbi.2023.104419.
10. Butler K, Bartlett YK, Newhouse N, Farmer A, French DP, Kenning C, et al. Implementing a text message–based intervention to support type 2 diabetes medication adherence in primary care: a qualitative study with general practice staff. *BMC Health Serv Res*. Jun 10, 2023;23(1):614. doi: 10.1186/s12913-023-09571-9.
11. Chiang Y-T, Chang C-W, Yu H-Y, Tsay P-K, Lo F-S, Chen C-W, et al. Developing the "Healthcare CEO App" for patients with type 1 diabetes transitioning from adolescence to young adulthood: a mixed-methods study. *Nurs Open*. Mar 2023;10(3):1755-1766. doi: 10.1002/nop2.1432.
12. De Luca V, Bozzetto L, Giglio C, Tramontano G, De Simone G, Luciano A, et al. Clinical outcomes of a digitally supported approach for self-management of type 2 diabetes mellitus. *Front Public Health*. 2023;11:1219661. doi: 10.3389/fpubh.2023.1219661.
13. Dening J, George ES, Ball K, Islam SMS. User-centered development of a digitally-delivered dietary intervention for adults with type 2 diabetes: the T2Diet Study. *Internet Interv*. Apr 2022;28:100505. doi: 10.1016/j.invent.2022.100505.
14. Ding H, Fatehi F, Russell AW, Karunanithi M, Menon A, Bird D, et al. User experience of an innovative mobile health program to assist in insulin dose adjustment: outcomes of a proof-of-concept trial. *Telemed J E Health*. Jul 2018;24(7):536-543. doi: 10.1089/tmj.2017.0190.
15. Duffy S, Norton D, Kelly M, Chavez A, Tun R, Ramírez M, et al. Using community health workers and a smartphone application to improve diabetes control in rural Guatemala. *Glob Health Sci Pract*. Dec 23, 2020;8(4):699-720. doi: 10.9745/GHSP-D-20-00076.
16. Fareed N, Swoboda C, Singh P, Boettcher E, Wang Y, Venkatesh K, et al. Developing and testing an integrated patient mHealth and provider dashboard application system for type 2 diabetes management among Medicaid-enrolled pregnant individuals based on a user-centered approach: mixed-methods study. *Digit Health*. 2023;9:20552076221144181. doi: 10.1177/20552076221144181.
17. Fico G, Martinez-Millana A, Leuteritz J-P, Fioravanti A, Beltrán-Jaunsarás ME, Traver V, et al. User-centered design to improve information exchange in diabetes care through eHealth: results from a small-scale exploratory study. *J Med Syst*. Nov 18, 2019;44(1):2. doi: 10.1007/s10916-019-1472-5.
18. Groat D, Soni H, Grando MA, Thompson B, Kaufman D, Cook CB. Design and testing of a smartphone application for real-time self-tracking diabetes self-management behaviors. *Appl Clin Inform*. Apr 2018;9(2):440-449. doi: 10.1055/s-0038-1660438.
19. Holtz BE, Murray KM, Hershey DD, Richman J, Dunneback JK, Vyas A, et al. The design and development of MyT1DHero: a mobile app for adolescents with type 1 diabetes and their parents. *J Telemed Telecare*. Apr 2019;25(3):172-180. doi: 10.1177/1357633X17745470.
20. Joachim S, Forkan ARM, Jayaraman PP, Morshed A, Wickramasinghe N. A nudge-inspired AI-driven health platform for self-management of diabetes. *Sensors (Basel)*. Jun 19, 2022;22(12):4620. doi: 10.3390/s22124620.
21. Kerfoot BP, Gagnon DR, McMahon GT, Orlander JD, Kurgansky KE, Conlin PR. A team-based online game improves blood glucose control in veterans with type 2 diabetes: a randomized controlled trial. *Diabetes Care*. Sep 2017;40(9):1218-1225. doi: 10.2337/dc17-0310.
22. Kilic M, Karadağ A. Developing and evaluating a mobile foot care application for persons with diabetes mellitus: a randomized pilot study. *Wound Manag Prev*. Oct 10, 2020;66(10):29-40. doi: 10.25270/wmp.2020.10.2940.
23. Krmpotic K, Gallant J, Zufelt K, Zuijdwijk C. User-centred development of an mHealth app for youth with type 1 diabetes: the challenge of operationalizing desired features and feasibility of offering financial incentives. *Health Technol*. Mar 15, 2022;12(2):499-513. doi: 10.1007/s12553-022-00656-9.
24. Leon N, Namadingo H, Bobrow K, Cooper S, Crampin A, Pauly B, et al. Intervention development of a brief messaging intervention for a randomised controlled trial to improve diabetes treatment adherence in sub-Saharan Africa. *BMC Public Health*. Jan 15, 2021;21(1):147. doi: 10.1186/s12889-020-10089-6.
25. Merino-Barbancho B, Barrera MJ, Vera-Muñoz C, Guirado JC, Arredondo MT, Fico G. Definition and development of a digital system for the empowerment and activation of type 1 diabetic patient. 2021. Presented at: 2021 43rd Annual International Conference of the IEEE Engineering in Medicine & Biology Society (EMBC); November 1-5, 2021:2230-2233; Mexico City, Mexico.
26. O'Reilly SL, Laws R. Health-e mums: evaluating a smartphone app design for diabetes prevention in women with previous gestational diabetes. *Nutr Diet*. Nov 2019;76(5):507-514. doi: 10.1111/1747-0080.12461.
27. Pais S, Petrova K, Parry D. Enhancing system acceptance through user-centred design: integrating patient generated wellness data. *Sensors (Basel)*. Dec 22, 2021;22(1):45. doi: 10.3390/s22010045.
28. Goyal S, Nunn CA, Rotondi M, Couperthwaite AB, Reiser S, Simone A, et al. A mobile app for the self-management of type 1 diabetes among adolescents: a randomized controlled trial. *JMIR Mhealth Uhealth*. Jun 19, 2017;5(6):e82. doi: 10.2196/mhealth.7336.
29. Petersen M, Hempler NF. Development and testing of a mobile application to support diabetes self-management for people with newly diagnosed type 2 diabetes: a design thinking case study. *BMC Med Inform Decis Mak*. Jun 26, 2017;17(1):91. doi: 10.1186/s12911-017-0493-6.
30. Pillalamarri SS, Huyett LM, Abdel-Malek A. Novel Bluetooth-enabled tubeless insulin pump: a user experience design approach for a connected digital diabetes management platform. *J Diabetes Sci Technol*. Nov 2018;12(6):1132-1142. doi: 10.1177/1932296818804802.
31. Ploderer B, Clark D, Brown R, Harman J, Lazzarini PA, Van Netten JJ. Self-monitoring diabetes-related foot ulcers with the MyFootCare app: a mixed methods study. *Sensors (Basel)*. Feb 24, 2023;23(5):2547. doi: 10.3390/s23052547.
32. Rodriguez DV, Lawrence K, Luu S, Yu JL, Feldthouse DM, Gonzalez J, et al. Development of a computer-aided text message platform for user engagement with a digital diabetes prevention program: a case study. *J Am Med Inform Assoc*. Dec 28, 2021;29(1):155-162. doi: 10.1093/jamia/ocab206.
33. Salari R, R Niakan Kalhori S, GhaziSaeedi M, Jeddi M, Nazari M, Fatehi F. Mobile-based and cloud-based system for self-management of people with type 2 diabetes: development and usability evaluation. *J Med Internet Res*. Jun 02, 2021;23(6):e18167. doi: 10.2196/18167.
34. Sinha AP, Singhal M, Joshi A, Kumar B, Sethiya NK. User-centered ‘Swayam Diabetes’m-health application for self-care management of type 2 diabetes in urban home settings: the usability and utility testing of mobile application and perspectives. *Int J Chem Biochem Sci*. 2023;24(5):141-146. doi: 10.35178/ijcbs.2023.024.018.
35. Smith R, Michalopoulou M, Reid H, Riches SP, Wango YN, Kenworthy Y, et al. Applying the behaviour change wheel to develop a smartphone application 'stay-active' to increase physical activity in women with gestational diabetes. *BMC Pregnancy Childbirth*. Mar 26, 2022;22(1):253. doi: 10.1186/s12884-022-04539-9.
36. Sobri NHM, Ismail IZ, Hassan F, Papachristou Nadal I, Forbes A, Ching SM, et al. MYGODDESS Project Team. Protocol for a qualitative study exploring the perception of need, importance and acceptability of a digital diabetes prevention intervention for women with gestational diabetes mellitus during and after pregnancy in Malaysia (Explore-MYGODDESS). *BMJ Open*. Aug 26, 2021;11(8):e044878. doi: 10.1136/bmjopen-2020-044878.
37. Steinman L, Heang H, van Pelt M, Ide N, Cui H, Rao M, et al. Facilitators and barriers to chronic disease self-management and mobile health interventions for people living with diabetes and hypertension in Cambodia: qualitative study. *JMIR Mhealth Uhealth*. Apr 24, 2020;8(4):e13536. doi: 10.2196/13536.
38. Sze WT, Waki K, Enomoto S, Nagata Y, Nangaku M, Yamauchi T, et al. StepAdd: a personalized mHealth intervention based on social cognitive theory to increase physical activity among type 2 diabetes patients. *J Biomed Inform*. Sep 2023;145:104481. doi: 10.1016/j.jbi.2023.104481.
39. Tanner AE, Palakshappa D, Morse CG, Mann-Jackson L, Alonzo J, Garcia M, et al. Exploring the consequences of food insecurity and harnessing the power of peer navigation and mHealth to reduce food insecurity and cardiometabolic comorbidities among persons with HIV: protocol for development and implementation trial of weCare/Secure. *Trials*. Dec 12, 2022;23(1):998. doi: 10.1186/s13063-022-06924-3.
40. Tay BSJ, Edney SM, Brinkworth GD, Cox DN, Wiggins B, Davis A, et al. Co-design of a digital dietary intervention for adults at risk of type 2 diabetes. *BMC Public Health*. Nov 11, 2021;21(1):2071. doi: 10.1186/s12889-021-12102-y
41. Waaler PN, Bongo LA, Rolandsen C, Lorem GF. Simplifying and personalising health information with mobile apps: translating complex models into understandable visuals. *medRxiv*. Preprint posted online in 2023 [doi: 10.1101/2023.05.25.23290511]. doi:10.1101/2023.05.25.23290511.
42. Yee LM, Leziak K, Jackson J, Strohbach A, Saber R, Niznik CM, et al. Patient and provider perspectives on a novel mobile health intervention for low-income pregnant women with gestational or type 2 diabetes mellitus. *J Diabetes Sci Technol*. Jul 05, 2020;15(5):1121-1133. doi: 10.1177/1932296820937347.
43. Zamanillo-Campos R, Fiol-deRoque MA, Serrano-Ripoll MJ, Mira-Martínez S, Ricci-Cabello I. Development and evaluation of DiabeText, a personalized mHealth intervention to support medication adherence and lifestyle change behaviour in patients with type 2 diabetes in Spain: a mixed-methods phase II pragmatic randomized controlled clinical trial. *Int J Med Inform*. Aug 2023;176:105103. doi: 10.1016/j.ijmedinf.2023.105103.
