# Supplementary material for: Adherence to Usability and Accessibility Principles in Digital Health Applications for Patients With Diabetes: Systematic Review
Source: J Med Internet Res. 2025 Sep 26;27:e71567. doi: 10.2196/71567 (PMC12514418; doi:10.2196/71567)
Supplement: Multimedia Appendix 3 [file jmir_v27i1e71567_app3.pdf]

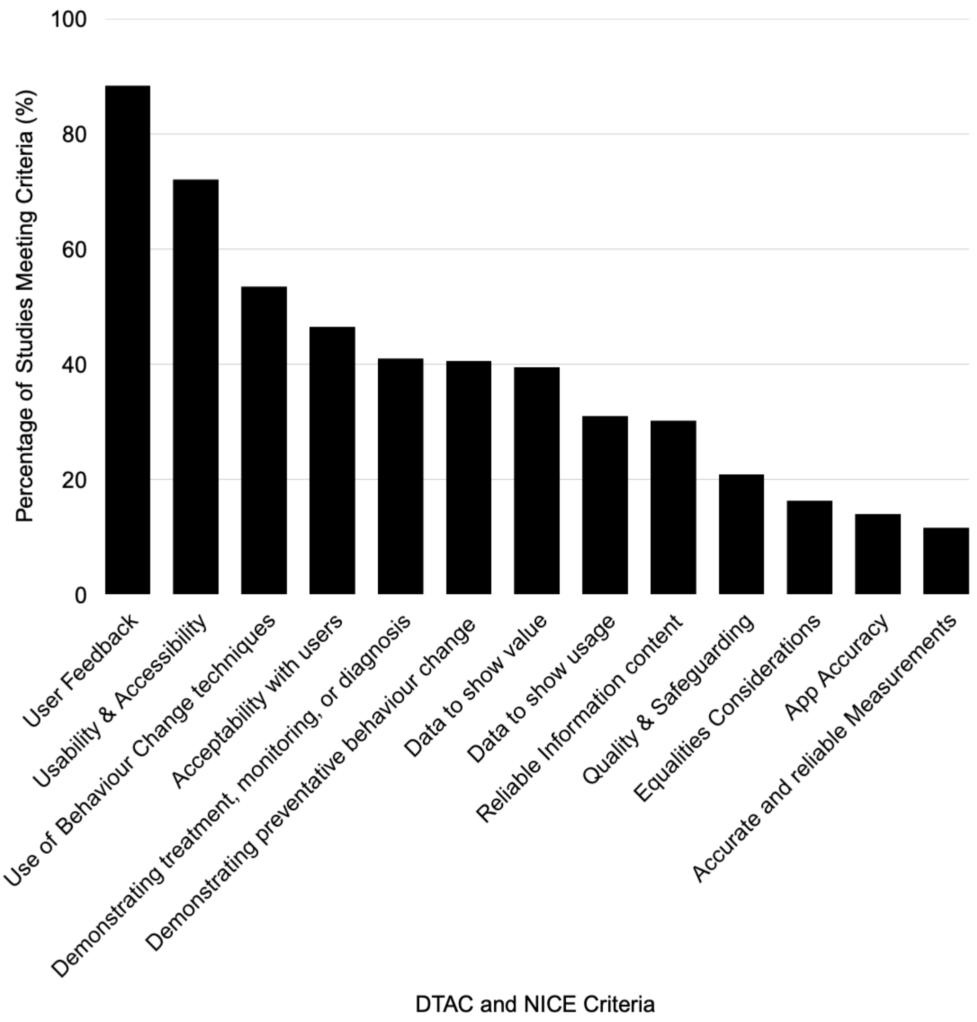

Multimedia Appendix 3. Bar chart showing the percentage of included studies (N=43) that met each of the 13 evaluated development criteria, grouped by domain (ie, user engagement, behavior change, monitoring), as derived from the DTAC 2021 and NICE 2022 digital health frameworks. DTAC: Digital Technology Assessment Criteria; NICE: National Institute for Health and Care Excellence.
